# Inequalities in cancer survival and the NHS cancer plan: evidence of progress?

**DOI:** 10.1038/sj.bjc.6605830

**Published:** 2010-08-10

**Authors:** S Harper

**Affiliations:** 1 Department of Epidemiology, Biostatistics and Occupational Health, McGill University, Montreal, Canada H3A 1A2

One reason for monitoring social inequalities in health is to evaluate whether the health effects of social or public health policies may differ by social position. The paper in this issue by Rachet *et al* (2010) aims to assess whether the NHS Cancer Plan has reduced social inequalities in cancer survival, and they generally conclude that it has not. There is much to commend in this study. The authors provide a comprehensive picture across more- and less-common cancers, disaggregated by gender and using up-to-date techniques for measuring cancer survival. They also measure relative survival using deprivation-specific life tables to estimate background mortality, which helps to guard against artifactual changes in survival inequalities that may arise if inequalities in background mortality are changing over time (Rachet *et al*, 2009). Their use of relatively small geographic units of analysis (approximately 1500 persons) to define deprivation as a proxy for individual socioeconomic position likely provides less misclassification than would larger areas such as electoral wards. In addition, although they measure relative survival as an outcome, in terms of inequalities in survival they present estimates of the absolute rather than the relative socioeconomic difference in survival rates. This latter point is important because secular trends in relative and absolute measures of health inequality often disagree (Moser *et al*, 2007), and absolute differences are typically seen reflecting the population health burden of inequalities.

Much more could be said about the positive aspects of this study (and the authors larger body of work on cancer surveillance) than its limitations, but a few methodological points are of interest. First is the issue of inequality measurement. Rachet and colleagues present only information on the survival trends of the most- and least-deprived quintiles of the population, which ignores the 60% of the population in the middle deprivation categories. One could argue that estimating the gap between the upper and lower quintile provides a legitimate summary of the extent of socioeconomic inequality, but knowing what's happening to the rest of the population also matters, as different patterns may be hidden by the same high-low gap, and may suggest different strategies for reducing inequalities. It is important to know, for example, whether the deprivation gap primarily results from a graded association between deprivation and survival, from an advantaged group that is doing considerably better than the rest of the population, or from a disadvantaged group that is doing considerably worse that the other 80% of the population. One could argue that the latter case may suggest a more troubling situation, if this indicates inadequate or lack of timely treatment (Raine *et al*, 2010). The point is that the range as a measure of inequality cannot identify these distinct patterns. I would encourage the authors in future work to complement their analysis of gaps using a summary measure of inequality that accounts for the full distribution of socioeconomic position and its association with cancer survival.

A second methodological limitation is the use of an ecological measure of deprivation as a proxy for individual-level information. The lack of individual-level measures of socioeconomic position necessitates this choice (unfortunately common in cancer surveillance), but it could potentially lead to underestimation of socioeconomic inequalities in survival. In the United States, for example, most cancer mortality gradients are considerably steeper when measured using individual rather than area-based socioeconomic indicators (Singh *et al*, 2003; Albano *et al*, 2007). And though trends in small-area deprivation inequality are likely to reflect the situation among individuals, the choice of indicator likely matters. Using longitudinal data from the Office of National Statistics, Sloggett *et al* (2007) found that the magnitude of the effect of socioeconomic position on cancer survival differed whether it was measured using individual or area-based indicators, though all indicators generally show poorer survival for the disadvantaged. The use of area-based indicators also by Rachet and colleagues also creates the potential for confounding by health-selected migration. If healthier individuals are more likely to move to less-deprived areas, this could potentially lead to increases in area-based health inequalities over time. The use of 1-year survival may make this less likely, but given evidence for this phenomenon for other health outcomes in the United Kingdom (Boyle, 2004; Cox *et al*, 2007) and elsewhere (Pearce and Dorling, 2010), it may be worth investigating further.

Third, it's worth emphasising that the estimates presented by Rachet and colleagues are model based, and thus subject to modelling assumptions. For good reason they attempted to estimate period-specific linear trends to isolate pre-Cancer plan, post-initialisation, and post-implementation differences. However, it's not clear whether this particular periodization provides a good fit to the data (there is also little discussion of how the periods of initialisation and implementation were distinguished). Commendably, they also experimented with a more flexible modelling strategy, but I disagree slightly with the authors that the trends and conclusions are identical using the two strategies. For example, using linear models Figure 2B supports their conclusion that the deprivation gap widened between 1996 and 2000 and then declined post-cancer plan, but the supplementary graph using a more flexible methods shows that the trends were constant over the entire period of study, and no change in inequality by period is evident. In the supplementary web table, the estimate of the change in the rectal cancer survival gap among women from 1996 to 2006 is a 0.7 percentage point increase in the gap when using a linear model, but a 0.8 percentage point decrease in the gap when measured using non-linear regression (the respective estimates for men are also different at 2.0 and 0.3). Similar differences in both the magnitude and the direction of change of the deprivation gap using linear versus non-linear models are evident for a number of other cancers (pancreas, bladder, melanoma, larynx). In short, although the modelling scheme may lead to generally similar conclusions for all cancers combined, for several specific cancers the results seem quite sensitive to model specification.

These methodological issues notwithstanding, I am inclined to agree with Rachet and colleagues that there is little evidence that the NHS Cancer Plan has yet to reduce inequalities in cancer survival. Very few of the estimates of the annual change in the deprivation gap for specific cancers for any one of the authors’ phases of the NHS plan statistically exclude zero (at conventional levels), and it seems unlikely that tests of whether the annual change differs between pre- and post-phases (arguably a better test of whether the plan is working) would show any effect. However, my view of the overall secular trend is somewhat more positive than that of the authors. If one ignores the potentially artificial designation between initialisation and implementation and looks at the entire period from 1996–2000 to 2004–2006, the results in Table 2 suggest that 21 of the 35 cancer-gender combinations are at least going in the right direction (i.e., decreasing inequality), though they are imprecisely estimated and may also reflect ceiling effects. Furthermore, inequalities in 1-year survival have improved for cancers with good prognosis for both men and women, and 3-year survival inequalities for both good and poor-prognosis cancers have improved for women. Rachet and colleagues are correct that this is no cause for celebration, as the magnitude of survival inequalities remains far too high to the disadvantage of poor areas, but one could nevertheless read these results as showing at least some progress toward success. This progress, however, seems largely the result of continued improvements in cancer survival for all social groups that have been occurring since at least the mid-1980s rather than the direct result of the NHS Cancer Plan. It may still be too early to tell whether the plan will ultimately succeed in reducing inequalities, but finding out will require the continuation of high-quality monitoring studies like that of Rachet and colleagues.
